# Immunomodulatory Properties and Osteogenic Activity of Polyetheretherketone Coated with Titanate Nanonetwork Structures

**DOI:** 10.3390/ijms23020612

**Published:** 2022-01-06

**Authors:** Yuanyuan Yang, Honghao Zhang, Satoshi Komasa, Tetsuji Kusumoto, Shinsuke Kuwamoto, Tohru Okunishi, Yasuyuki Kobayashi, Yoshiya Hashimoto, Tohru Sekino, Joji Okazaki

**Affiliations:** 1 Department of Removable Prosthodontics and Occlusion, Osaka Dental University, 8-1 Kuzuha-hanazono-cho, Hirakata 573-1121, Japan; yangyuanyuan0801@outlook.com (Y.Y.); komasa-s@cc.osaka-dent.ac.jp (S.K.); joji@cc.osaka-dent.ac.jp (J.O.); 2 Faculty of Health Sciences, Osaka Dental University, 1-4-4, Makino-honmachi, Hirakata-shi, Osaka 573-1144, Japan; kusumoto@cc.osaka-dent.ac.jp; 3 OIKE & Co., Ltd., 181 Tokusayama-cho, Nishinotoin Nishi-iru, Bukkoji, Shimogyo-ku, Kyoto 600-8461, Japan; s-kuwamoto@oike-jp.com (S.K.); t-okunishi@oike-jp.com (T.O.); 4 Osaka Research Institute of Industrial Science and Technology Morinomiya Center, 1-6-50, Morinomiya, Joto-ku, Osaka 536-8553, Japan; kobaya@omtri.or.jp; 5 Department of Biomaterials, Osaka Dental University, 8-1 Kuzuhahanazono-cho, Hirakata-shi, Osaka 573-1121, Japan; yoshiya@cc.osaka-dent.ac.jp; 6 SANKEN—The Institute of Scientific and Industrial Research, Osaka University, Ibaraki, Osaka 567-0047, Japan; sekino@sanken.osaka-u.ac.jp

**Keywords:** PEEK, nanostructure, immune response, osteoimmunomodulation, osteogenic activity

## Abstract

Polyetheretherketone (PEEK) is a potential substitute for conventional metallic biomedical implants owing to its superior mechanical and chemical properties, as well as biocompatibility. However, its inherent bio-inertness and poor osseointegration limit its use in clinical applications. Herein, thin titanium films were deposited on the PEEK substrate by plasma sputtering, and porous nanonetwork structures were incorporated on the PEEK surface by alkali treatment (PEEK-TNS). Changes in the physical and chemical characteristics of the PEEK surface were analyzed to establish the interactions with cell behaviors. The osteoimmunomodulatory properties were evaluated using macrophage cells and osteoblast lineage cells. The functionalized nanostructured surface of PEEK-TNS effectively promoted initial cell adhesion and proliferation, suppressed inflammatory responses, and induced macrophages to anti-inflammatory M2 polarization. Compared with PEEK, PEEK-TNS provided a more beneficial osteoimmune environment, including increased levels of osteogenic, angiogenic, and fibrogenic gene expression, and balanced osteoclast activities. Furthermore, the crosstalk between macrophages and osteoblast cells showed that PEEK-TNS could provide favorable osteoimmunodulatory environment for bone regeneration. PEEK-TNS exhibited high osteogenic activity, as indicated by alkaline phosphatase activity, osteogenic factor production, and the osteogenesis/osteoclastogenesis-related gene expression of osteoblasts. The study establishes that the fabrication of titanate nanonetwork structures on PEEK surfaces could extract an adequate immune response and favorable osteogenesis for functional bone regeneration. Furthermore, it indicates the potential of PEEK-TNS in implant applications.

## 1. Introduction

Polyetheretherketone (PEEK) is a prime candidate of alternative biomaterial to traditional metallic implants made of titanium and its alloys, because PEEK exhibits superior mechanical properties (especially elastic modulus), high chemical resistance, inherent radiolucency, biocompatibility, and stability in vivo [1,2,3]. The use of titanium implants has been highly successful in clinical applications. However, the release of superfluous ions and the stress shielding that leads to implant failure is a major concern [4,5,6]. The large elastic modulus (>100 GPa) of titanium implants causes frequent bone absorption. However, the elastic modulus of PEEK (3–4 GPa) is approximately equal to the elastic modulus of human bone. This reduces bone absorption and osteoporosis caused by stress shielding [7,8]. PEEK has been increasingly employed for spinal fusion cages, artificial knee joints, fixation plates and screws, and neurosurgical/craniomaxillofacial prostheses since the 1990s, when the first implantable grade PEEK was developed commercially [9]. However, the lack of surface bioactivity and osseointegration resulting in fibrous encapsulation around PEEK implants hinders their orthopedic applications [10,11].

Researchers have used various methods of imparting biological activity to the surface of PEEK to improve the binding between PEEK and bone. These methods can be divided into three types [12]: incorporation of bioactive agents directly on the surface of PEEK [13]; surface functionalization with bioactive agents using either physical or chemical methods [14]; and in situ manufacturing of macro-, micro-, or nanoporous structures [15]. Bioactive agent coatings by direct incorporation or surface functionalization exhibit excellent osteoconductive capability. However, bioactive agent coatings precipitate and wear over time. Moreover, the brittleness and relatively poor mechanical properties of the coatings limit their clinical application to non-load-bearing implants [16]. Modification of the surface morphology gives PEEK unique surface characteristics and improves the hydrophilicity of the surface that has a direct impact on the initial adhesion of bone marrow stem cells to the surface and subsequent proliferation and differentiation behavior [17]. Several researchers have established that the biocompatibility of PEEK stably increases over a long duration by modifying the surface morphology using different methods, such as vacuum evaporation, acid etching, and sulfonation [18,19,20,21,22,23,24,25,26]. However, these methods of surface morphology modification are time-consuming and require large machines or high costs, making their clinical application difficult. In recent years, titanium (Ti) coating on the surface of PEEK has become a highly feasible and widely used surface topography modification method [27,28,29,30,31,32,33,34,35,36]. Ti exhibits high bone conductivity, and the Ti coating can be given a special porous nanostructure morphology in multiple simple ways. High-concentration alkali treatment at room temperature is a low-energy, reliable, and cost-effective morphology modification technology. High-concentration alkali treatment at room temperature can effectively form a porous nanoscale network structure, and in vitro and in vivo experiments have demonstrated that it can improve osteogenic activity and bone formation [37,38,39,40]. Therefore, high-concentration alkali treatment at room temperature is an easy and fast method for manufacturing highly biocompatible dental and orthopedic implants with nanonetwork structures.

A blood clot forms around an implant in a bone first. Moreover, the local trauma of the implantation operation encourages immune cells, such as macrophages and T cells, to concentrate around the implant because of local immunity inflammation. Furthermore, bone marrow-derived mesenchymal stem cells attach to the surface of the implant and begin the osteogenic differentiation process. The impact of the implant on attached macrophages and the reciprocal interaction between immune cells and bone marrow mesenchymal stem cells make osseointegration of the implant successful [41,42,43,44,45]. Osteoimmunology focuses on the close correlation between the immune-inflammatory reaction that occurs soon after the implantation of biomaterials into a human body and the subsequent bone growth process [46,47,48]. The dynamic balance between bone resorption and bone formation guided by immune cells is the most important factor in maintaining bone constancy and bone remodeling. The existing implant biomaterials require stronger bone formation than bone resorption. Therefore, the amount of bone around the bio-implant material continues to increase, and excessive inhibition of bone resorption can lead to low-quality bone formation. Macrophages are the immune cells that reach the wound site immediately after bio-implantation materials are implanted in a human body. Macrophages release cytokines, chemical factors, and other substances to guide the recruitment, proliferation, and differentiation of bone marrow mesenchymal stem cells by immune-inflammatory responses in the local area [49,50]. Macrophages differentiate into M1 and M2 cells based on the characteristics of the implant surface after attaching to the surface. M1 cells secrete interleukin 6 (IL-6), tumor necrosis factor α (TNFα), and other proinflammatory cytokines to strengthen local inflammation, whereas M2 cells secrete interleukin 10 (IL-10), bone morphogenetic protein 2 (BMP-2), vascular endothelial growth factor (VEGF), and other pro-tissue repair factors to reduce the local inflammation levels and promote tissue regeneration [51,52].

We deposited a thin titanium coating on the surface of PEEK by sputtering and produced porous nanonetwork structures by high-concentration alkali treatment at room temperature. Moreover, we observed that the modification of the PEEK surface with nanonetwork structures and changes in its chemical properties can significantly improve the biocompatibility of PEEK and accelerate the process of bone formation. We analyzed the ability of the immune response of macrophages and the interaction between macrophages and MSCs to enhance osseointegration. The study showed that the fabrication of titanate nanonetwork structures on PEEK surfaces could extract an adequate immune response and favorable osteogenesis for functional bone regeneration. Furthermore, it indicates the potential of PEEK-TNS in implant applications.

## 2. Results

### 2.1. Surface Characterization

**Figure 1:**
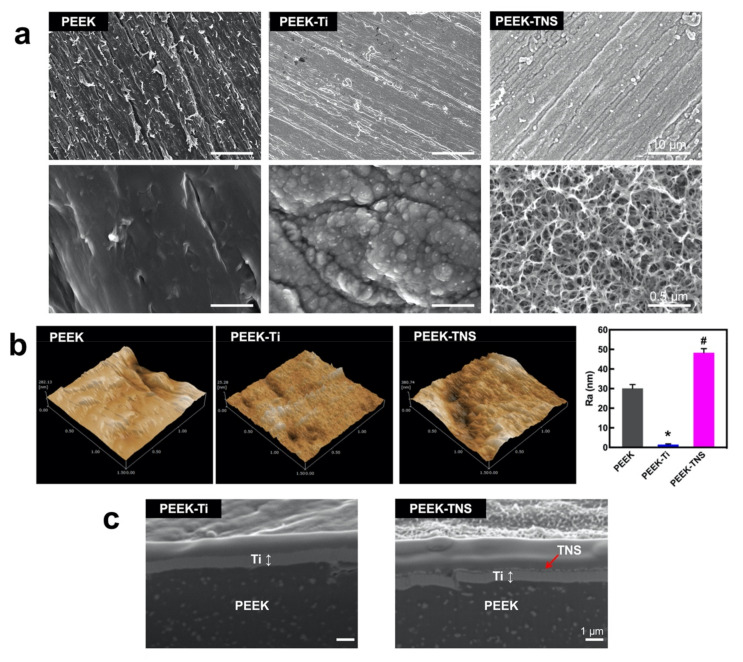
The surface morphology of PEEK, PEEK-Ti, and PEEK-TNS. (**a**) SEM images, scale bar = 10, 0.5 µm. (**b**) Three-dimensional surface topography detected by SPM and the mean average surface roughness (Ra). (**c**) Cross-sectional morphology of PEEK-Ti and PEEK-TNS, scale bar = 1 µm. (* represents *p* < 0.05 when PEEK-Ti is compared with PEEK; # represents *p* < 0.05 when PEEK-TNS is compared with PEEK-Ti).

The surface morphologies of PEEK, PEEK-Ti, and PEEK-TNS are illustrated in Figure 1a. The SEM analysis detected several scratches on the PEEK surface due to pre-polishing. PEEK-Ti exhibited a relatively flat and smooth surface with accumulated Ti particles distributed on the substrates after plasma sputtering with titanium. As shown in the high-resolution image of PEEK-TNS, a homogeneous nanoporous network structure was produced on the surface after alkali treatment. The topographical features were validated using SPM (Figure 1b). PEEK-Ti exhibited the lowest surface roughness, whereas PEEK-TNS exhibited the highest surface roughness, resulting in the modulation of cell behavior. The cross-sectional morphology (Figure 1c) demonstrates that the Ti coating layer was tightly bonded to the PEEK surface, and no distinct changes were observed after alkali treatment.

**Figure 2:**
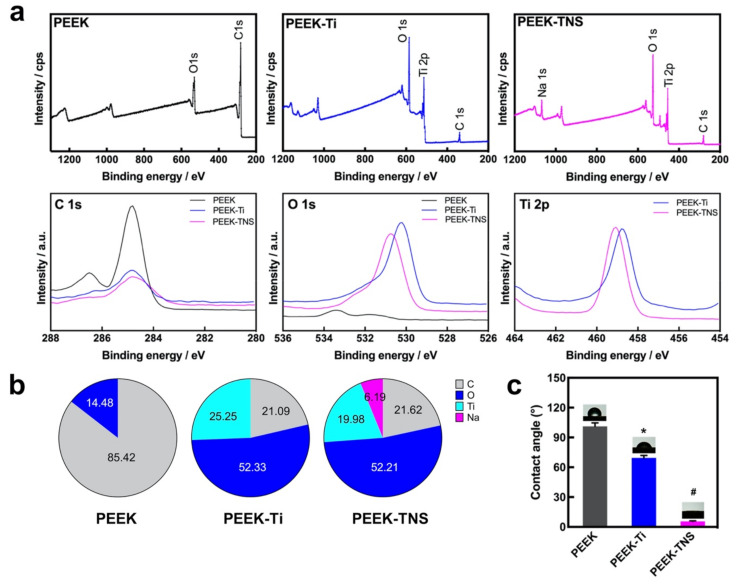
Surface chemical characteristics of PEEK, PEEK-Ti, and PEEK-TNS. (**a**) XPS survey spectra of PEEK, PEEK-Ti and PEEK-TNS by a wide analysis, and XPS survey spectra of C 1s, O 1s, and Ti 2p peaks. (**b**) Elemental composition. (**c**) Water contact angles. (* represents *p* < 0.05 when PEEK-Ti is compared with PEEK; # represents *p* < 0.05 when PEEK-TNS is compared with PEEK-Ti).

The surface chemical compositions of PEEK, PEEK-Ti, and PEEK-TNS were also analyzed using XPS, as shown in Figure 2a. Only the C 1s and O 1s peaks were detected on the PEEK surface, whereas the Ti 2p peak appeared on the PEEK-Ti surface. Moreover, the appearance of a Na 1s peak on the PEEK-TNS surface demonstrates that sodium is present on the titanium film surface due to the high-concentration alkali treatment. Furthermore, the C1s peak and the atomic percentage of carbon decreased in PEEK-Ti and PEEK-TNS, whereas the atomic percentage of oxygen increased significantly, resulting in better wettability and biocompatibility.

The surface hydrophilicity of the samples was determined by the contact angle measurements. The water contact angle for PEEK was 100.4° and decreased to 67.9° after the deposition of Ti films on the PEEK surface. Moreover, the contact angle of the PEEK-TNS surface decreased significantly to 5.5° after treatment with alkali. The changes in the contact angles were caused due to the transformation of the surface physicochemical properties.

### 2.2. Inflammatory Response of Macrophages

#### 2.2.1. Cell Morphology and Viability of Macrophages

**Figure 3:**
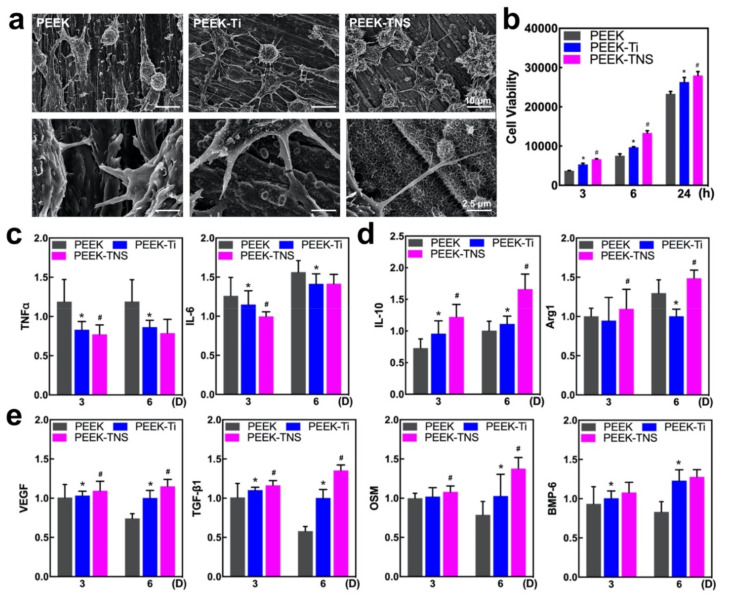
The immune response of RAW 264.7 on different samples of PEEK, PEEK-Ti, and PEEK-TNS, scale bar = 10, 2.5 µm. (**a**) SEM images illustrating cell growth and morphology of RAW 264.7. (**b**) Cell proliferation for 3, 6, and 24 h. (**c**) RT-qPCR of the M1-related genes expressions. (**d**) RT-qPCR of the M2-related genes expressions. (**e**) RT-qPCR of the osteogenic-related gene expressions. (* represents *p* < 0.05 when PEEK-Ti is compared with PEEK; # represents *p* < 0.05 when PEEK-TNS is compared with PEEK-Ti).

The cell morphology of the macrophages was determined using SEM after 24-h incubation. As shown in Figure 3a, the cells attached and spread efficiently on all tested surfaces. The macrophages exhibited an elongated spindle shape, along with the scratches on the PEEK surface. The macrophages grown on PEEK-Ti surfaces exhibited a spherical morphology. Moreover, the PEEK-TNS surface after the alkali treatment effectively changed the cell shapes and made the cells round with apparent elongated pseudopodia that tended to adhere to nanopores. The cell viability results after culturing for 3, 6, and 24 h were demonstrated in Figure 3b. The PEEK-TNS group exhibited the highest cell viability at both time points.

#### 2.2.2. Polarization of Macrophages

The expression of the M1- and M2-type-related genes was determined using RT-qPCR after culturing for 3 and 6 days to analyze the impacts of different samples on macrophage polarization (Figure 3c,d). Compared with PEEK, the proinflammatory genes (M1-related genes, such as TNF-α and IL-6) were significantly downregulated in PEEK-Ti and PEEK-TNS, whereas the anti-inflammatory genes (M2-related genes, including IL-10 and Arg-1) were upregulated on the PEEK-TNS surface. This demonstrates that PEEK-TNS possesses the ability to modulate the polarization of macrophages that inhibit proinflammatory M1 polarization and promote anti-inflammatory M2 polarization.

#### 2.2.3. Osteogenic Activities of Macrophages

The level of osteogenic gene expression in macrophages was assessed using RT-qPCR to define the osteoimmune environment on different surfaces exhaustively. As shown in Figure 3e, all four types of osteogenic gene expressions were upregulated on PEEK-Ti and PEEK-TNS. The fold changes in the osteogenic gene expression were considerably low when the culturing time was increased to 6 days, whereas the PEEK-TNS surface tended to secrete high levels of osteogenic factors as the culture time increased. These factors are highly important for generating an osteogenic microenvironment for bone regeneration.

### 2.3. Osteogenic Differentiation of rBMMSCs Induced by Macrophages

#### 2.3.1. Osteogenesis and Osteoclastogenesis-Related Gene Expression of rBMMSCs

**Figure 4:**
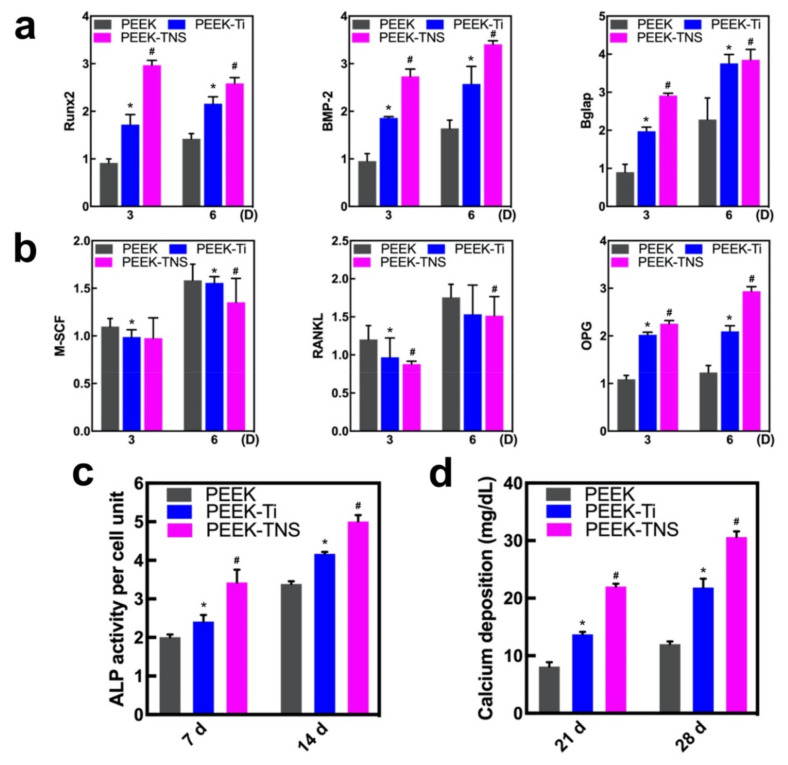
Osteogenic differentiation of rBMMSCs induced by macrophages on the PEEK, PEEK-Ti, and PEEK-TNS samples. (**a**) RT-qPCR of the osteogenic-related gene expressions. (**b**) RT-qPCR of the osteoclastogenesis-related gene expressions. (**c**) ALP activity levels of days 7 and 14. (**d**) Mineralization levels of days 21 and 28. (* represents *p* < 0.05 when PEEK-Ti is compared with PEEK; # represents *p* < 0.05 when PEEK-TNS is compared with PEEK-Ti).

The expression of osteogenic genes (Runx2, BMP-2, and Bglap) in rBMMSCs cultured with different CM for 3 and 6 days was determined using RT-qPCR (Figure 4a). The PEEK-TNS groups demonstrated a high expression of all the genes at both time points, indicating that the PEEK-TNS coculture system exhibited the highest capacity to promote the osteo-differentiation of rBMMSCs. Moreover, the expression of the osteoclastogenic genes M-SCF and RANKL was significantly downregulated in PEEK-TNS in a time-dependent manner (Figure 4b). However, OPG expression that inhibited the regulation of osteoclast differentiation was upregulated in the PEEK-TNS group. Therefore, PEEK-TNS may modulate the immune response that favors osteogenesis for functional bone regeneration.

#### 2.3.2. Alkaline Phosphatase (ALP) Activity and ECM Mineralization of rBMMSCs

The ALP activity of the rBMMSCs was assayed after osteogenic induction for 7 and 14 days (Figure 4c). Compared to PEEK, PEEK-Ti upregulated ALP activities that were further increased by PEEK-TNS. Moreover, the results of ECM mineralization (Figure 4d) exhibited a similar trend compared to the trend of ALP activity. Therefore, PEEK-TNS exhibits a high osteogenic differentiation of rBMMSCs by modulating the osteoimmune environment.

### 2.4. Osteogenic Differentiation of rBMMSCs on Different Samples

#### 2.4.1. Cell Morphology and Viability of rBMMSCs on Different Samples

**Figure 5:**
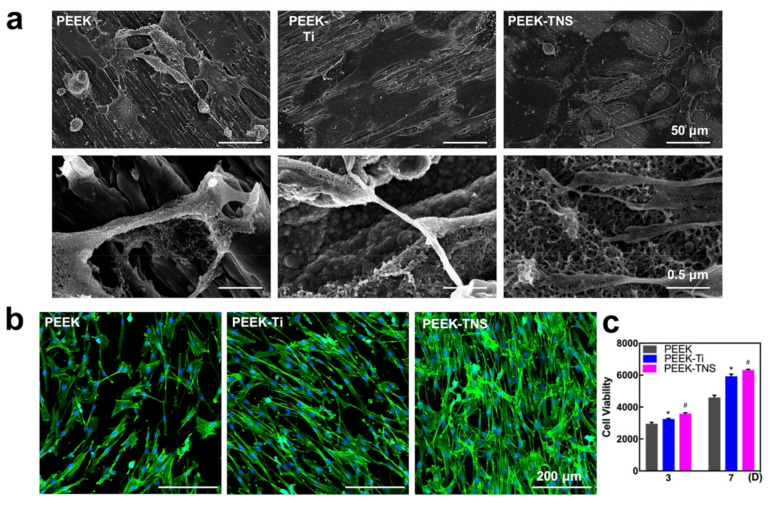
Cell growth, morphology, and proliferation of rBMMSCs on PEEK, PEEK-Ti, and PEEK-TNS. (**a**) SEM images illustrating the cell growth and morphology of rBMMSCs. (**b**) Cytoskeletons of rBMMSCs stained with phalloidin (green) and nuclei stained with DAPI (blue) for 24-h culturing. (**c**) Cell proliferation for 3 and 7 days. (* represents *p* < 0.05 when PEEK-Ti is compared with PEEK; # represents *p* < 0.05 when PEEK-TNS is compared with PEEK-Ti).

RBMMSC attachment and proliferation of the samples were analyzed after incubation for 24 h. As shown in Figure 5a, the cells on the PEEK surface exhibited few pseudopodia extensions. The cells cultured on the PEEK-Ti surface extended farther but still attached poorly to the samples with short pseudopodia extensions. However, the cells on the PEEK-TNS surfaces stretched and stacked with a polygonal geometry, as well as exhibited long filopodia, indicating a close connection to the nanopores of the surface. Figure 5b illustrates the images of actin cytoskeletal organization (green, labeled with Alexa Fluor^®^ 488 Phalloidin) and nuclei morphology (blue, stained by DAPI dye solution) of rBMMSCs adhering to the various samples. Cells on the PEEK-TNS surface occupied nearly the entire substrate surface and exhibited several filopodia and lamellipodia with abundant pseudopods to interconnect with each other. The cell proliferation was measured using Cell Titer Blue, as shown in Figure 5c. The PEEK-TNS groups exhibited significantly higher cell proliferation than the PEEK-Ti and PEEK groups. This was consistent with the observations from confocal laser scanning microscopy (CLSM).

#### 2.4.2. Osteogenic Behavior of rBMMSCs on Different Samples

**Figure 6:**
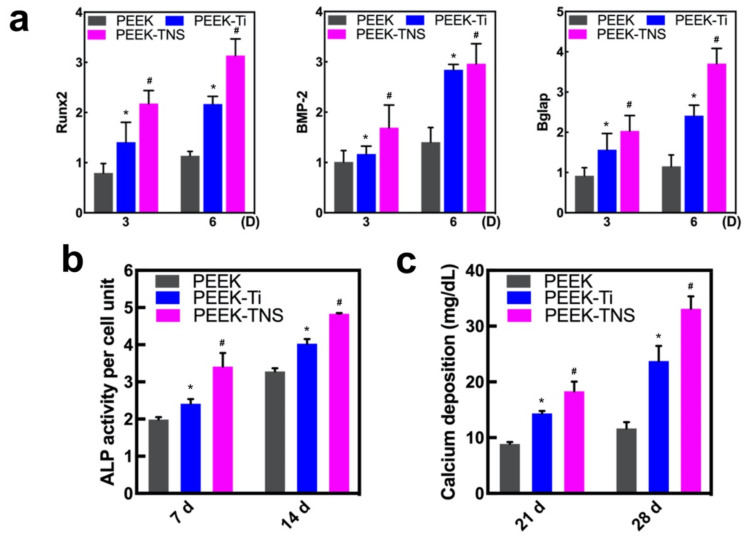
Osteogenic differentiation of rBMMSCs on PEEK, PEEK-Ti, and PEEK-TNS. (**a**) RT-qPCR of the osteogenic-related gene expressions. (**b**) ALP activity levels of days 7 and 14. (**c**) Mineralization levels of 21 and 28 days. (* represents *p* < 0.05 when PEEK-Ti is compared with PEEK; # represents *p* < 0.05 when PEEK-TNS is compared with PEEK-Ti).

The osteogenesis of rBMMSCs cultured on PEEK, PEEK-Ti, and PEEK-TNS was evaluated with respect to the osteogenic gene expressions, ALP activity, and ECM mineralization, as shown in Figure 6. The gene expression of RUNx2 and Bglap of the rBMMSCs on PEEK-TNS was significantly higher than those on PEEK and PEEK-Ti (Figure 6a). The results of Figure 6b,c demonstrated that the ALP activity and ECM mineralization of PEEK-TNS were the highest.

## 3. Discussion

In this study, thin titanium films were successfully deposited on the PEEK substrate, and porous nanonetwork structures were then fabricated on the PEEK surface by alkali treatment (PEEK-TNS). The surface physical and chemical characteristics were tested by SEM, XPS, and water contact angle measurements. We primarily focused on the osteoimmune and osteogenic efficiencies of PEEK coated with a titanate nanonetwork structure by analyzing the cell attachment, proliferation, M1/M2 polarization, osteogenesis/osteoclastogenesis-related gene expression of macrophages, and the osteogenesis ability of bone marrow mesenchymal cells that were grown in the conditioned environment by macrophages. From the SEM, the macrophages on PEEK-TNS exhibited a flat and elongated spindle shape with a larger attachment area. Macrophages grown on the surface of PEEK-TNS can proliferate and differentiate into M2 cells quickly and promote the repair of trauma tissue around PEEK-TNS. Moreover, the results of the osteogenesis-related gene expression, ALP activity, and mineralization levels of the rBMMSCs exhibited that the titanium nanonetwork structure can manipulate macrophages to increase osteogenic differentiation and bone formation in bone marrow mesenchymal cells. This demonstrates the significance of the titanium nanostructure coating on PEEK.

The interface between biomaterials surface and cells is essential for a variety of cellular behaviors [53,54,55]. The hydrophilicity and surface morphology of the implant surface have a significant impact on the biocompatibility and local inflammation control that ultimately determines the quality of implant osseointegration [56]. Several researchers have proven that implants with high hydrophilicity and nano-rough surface topography can provide long-term stable osseointegration of the implant using the interlocking effect with bone and by providing new bone with high bonding strength between the newly formed bone and the implant [57,58,59]. In our research, the contact angle of PEEK was reduced from 100° to 67° after titanium coating, and the PEEK-TNS achieved near super-hydrophilicity (Figure 2c), which could promote higher adsorption of fibronectin and earlier gene response for cell adhesion and cell differentiation [60,61]. Furthermore, during the alkali treatment of PEEK-Ti, we could determine that the Ti-O-Ti bonds were broken by the treatment with NaOH solution and then generated Ti-O-Na and Ti-OH bonds. It not only changed the physical properties of the biomaterials to nano-structures with a greater Ra but, at the same time, the changes in the chemical properties had more profound impacts on cell functions [62]. The PEEK-TNS surface can lead a large amount of blood protein to attach to the surface at the moment of implantation [63]. These proteins quickly spread throughout the surface of PEEK-TNS, which is extremely important for the subsequent formation of blood clots. Moreover, the nanostructure of TNS enhances osteogenic differentiation, because TNS is a loose nanoporous structure that can capture various cytokines and adhesion-promoting proteins in the blood. The nanoporous structure could provide more space for the pseudopodia of the bone marrow mesenchymal stem cells to penetrate deeply into the nanopores and hold tightly, allowing the cells to complete the attachment process quickly and begin to proliferate [37,38]. Furthermore, the cytokines and proteins can aggregate and store in the loose nanopores and continuously enhance the recruitment and attachment of bone marrow mesenchymal cells. Therefore, PEEK-TNS can promote cell adhesion and promote the proliferation and differentiation of bone marrow mesenchymal stem cells at the genetic level.

We used RAW 264.7 cells to observe the different behaviors and cellular responses of immune cells to the PEEK and PEEK-TNS surfaces. The SEM results demonstrated that PEEK-TNS had more attached macrophages compared to the macrophages attached to PEEK. Moreover, the cells were more strongly stretched and more evenly distributed on the surface of PEEK-TNS than on the surface of PEEK (Figure 3a). PEEK-Ti and PEEK-TNS significantly promoted the proliferation of macrophages and reduced the expression levels of inflammation-related genes (IL-6 and TNF-α) in the macrophages (Figure 3c) [64]. The expression level of the genes related to the M2 differentiation of macrophages (IL-10 and Arg1) [65] on PEEK-TNS was higher than the expression on PEEK. This demonstrates that PEEK-TNS can guide macrophages to differentiate into M2 cells and promote tissue repair and healing (Figure 3d). TNF-α and IL-6 can regulate cell death in inflamed tissues, change the permeability of the vascular endothelium, and induce the production of C-reactive protein in the acute phase. TNF-α and other inflammatory cytokines cause transient activation of TNF-α and Janus kinase-signal transducers and activators of transcription (JAK-STAT) signaling pathways and contribute to the high expression of transcription factors (such as NF-κB) [66,67,68,69,70]. From the results of the gene expression, macrophages grown on PEEK-TNS exhibited stronger angiogenesis (VEGF), fibroblast transformation (TGF-β1), and osteogenic differentiation (BMP-6, OSM) induction ability (Figure 3e) [71,72]. Omar et al. demonstrated that macrophages recognize and penetrate the fibrin scaffold formed on the implant surface after implantation by the CD163 cell surface marker. The M2 phenotype cells carrying CD163 are called myeloid-derived suppressor cells (MDSCs). MDSCs are derived from the bone marrow and regulate inflammation by inhibiting T-cell activity. In addition, MDSCs promote vascularization and produce a series of growth factors to promote rapid wound healing [73]. From the investigation of macrophages on different surfaces, we found that PEEK-TNS implants tend to promote the alleviation of tissue inflammation, tissue repair, and fast callus transformation into bone tissue. In our future research, we shall attempt to create a layer of fibrin scaffold on the surface of PEEK-TNS before macrophages are cultured to observe whether macrophages can exhibit fast attachment, proliferation speed, and M2 differentiation ability without direct contact.

Mere facilitation of the proliferation and differentiation of macrophages into M2 cells is not sufficient to prove the positive impact of PEEK-TNS on bone formation in bone marrow mesenchymal cells. It is of great significance for biomaterials to guide adequate osteoimmune modulation of macrophages cells and find out the optimize macrophage-osteoblast cells crosstalk for bone regeneration. Therefore, we used a conditioned medium containing the secretion of macrophages to cultivate bone marrow mesenchymal stem cells to simulate the bone marrow environment around the implant. The conditioned medium collected from macrophages cultured on PEEK-TNS showed the strongest impacts on promoting osteogenesis compared to the impact of the medium collected from macrophages cultured on PEEK and PEEK-Ti with respect to the proliferation of bone marrow mesenchymal stem cells, the expression of osteogenesis-related genes, ALP activity, and the amount of calcium deposition (Figure 4). Therefore, PEEK-TNS implants can guide macrophages to produce more osteogenic cytokines that aid in osteogenic differentiation after implantation, shortening the osteogenic process and providing high bone mass, as well as bone quality. In addition, the bone tissue formation around the implant undergoes two processes simultaneously after implantation into the bone: resorption of old bone tissue and establishment of new bone tissue. The combined effects of these two processes determines the rate at which the implant can reach stable osseointegration. The resorption of old bone tissue and the formation of new bone tissue reaches a balance in the fourth week after implantation and the initial stability of the implant is at its lowest level (the old bone has been absorbed but the new bone is not yet mature so that the bone cannot provide a strong fixation for the implant) [74]. This bone remodeling process performed by osteoclasts and osteoblasts is induced by macrophages [75]. The rapid new bone formation can aid the implant to obtain a better fixation of the new bone origin during the most dangerous period, reducing the possibility of early failure of the implant. PEEK-TNS can hasten the osteogenesis of the bone remodeling process so that the most dangerous period of the implant can occur early, and the osseointegration of the implant can be quick. Taken together, these findings suggest that the osteoimmune environment modulated by PEEK-TNS significantly enhanced the osteogenic differentiation of bone marrow mesenchymal cells.

**Figure 7:**
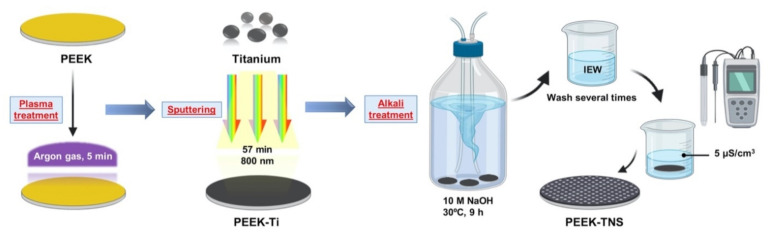
Schematic illustration of the PEEK-Ti and PEEK-TNS sample processing procedures. Prior to titanium film deposition, the PEEK substrates underwent argon plasma treatment at 30 W and 60 Pa for 5 min. The titanium films of 800 nm thickness were then deposited on the plasma-treated PEEK by the plasma sputtering process. The titanium-coated PEEK samples were denoted by PEEK-Ti. The PEEK-Ti samples were immersed in 10-M NaOH at 30 °C for 9 h and rinsed several times with ion-exchanged water until the solution reached a conductivity of <5 μS/cm^3^ for the fabrication of porous nanonetwork structures on the surface. The PEEK samples with porous nanonetwork structures were denoted by PEEK-TNS.

During our research, we also observed that the rate of rBMMSC proliferation and osteogenic differentiation on PEEK-TNS were increased (Figure 5 and Figure 7), contrary to the observations by researchers in the past [57,76,77,78]. We believe this is because PEEK-TNS has a nano-level morphology and not a micro-level morphology. Researchers in the past have demonstrated that the micro-level titanium topographies mimic the resorption pockets of osteoclasts in the natural state and have an impact on osteoblasts, as well as reduce early ALP activity and late calcium deposition. The titanium-coating technology used in our research can reduce the average roughness of the titanium layer on the PEEK-Ti surface to 1.7 nm. No micro-level morphology exists on the surface after alkali treatment, and the roughness can be increased to approximately 50 nm. Osteoblasts on the surface are only affected by the nanoscale morphology. From the SEM images of the bone marrow mesenchymal stem cells (Figure 5a), we observe that the pseudopodia of the bone marrow mesenchymal stem cells on PEEK-TNS can stretch long, and its tip can stretch into the nanoporous structure and tightly grasp the surface of the PEEK-TNS. This is conducive to the process of osteogenic differentiation. Osteoblasts on the surface of pure titanium grow as the following principle: the rate of osteoblast proliferation and differentiation is negatively correlated; that is, when the proliferation activity of osteoblasts is strong, their differentiation slows down and vice versa. This is partly because opposite growth factors regulate osteoblast proliferation and differentiation [79,80,81,82]. Nevertheless, our previous studies have shown that this principle does not apply to titanium surfaces with nano-topography. Both proliferation and differentiation can be improved simultaneously using TNS on pure titanium, as well as on a PEEK surface. We believe that the growth factors that alter the proliferation and differentiation of osteoblasts growing on microtopography have no impact on the proliferation and differentiation of the osteoblasts that grow on the surface of the nano-topography or other cytokines with the stronger growth promotion impact their proliferation and differentiation. This will be our future research topic.

Detailed research on the osteoimmune effects of dental and orthopedic implant materials is still inadequate. Classical implant material studies primarily analyze bone marrow mesenchymal stem cells, osteoblasts, and fibroblasts. However, research methods for studying immune-derived cells, such as macrophages and T cells, are still being developed. Another worthy research topic is osteoclasts [46,83,84,85]. This is because an analysis of the process by which osteoclasts dissolve old bone and low-quality new bone is important for the formation of high-quality new bone during bone remodeling. Our research will be the foundation for further studies on the impact of PEEK-TNS on future bone remodeling processes.

## 4. Materials and Methods

### 4.1. Surface Preparation

Medical grade Φ10-mm × 4-mm PEEK disks were supplied by Mitsubishi Chemical Corporation (Tokyo, Japan). The samples were ground progressively with sandpaper up to 1500 grit, as well as ultrasonically cleaned with acetone, ethanol, and deionized water for 10 min separately for PEEK control. The PEEK substrates underwent argon plasma treatment at 30 W and 60 Pa for 5 min before titanium film deposition. The 800 nm thick titanium films were deposited on the plasma-treated PEEK by plasma sputtering (OIKE & Co., Ltd., Kyoto, Japan) at 250 W and 9.5 × 10^−2^ Pa for 57 min. The titanium-coated PEEK substrates are denoted by PEEK-Ti. For the fabrication of porous nanonetwork structures on the sample surface, PEEK-Ti were immersed in 10-M NaOH at 30 °C for 9 h and rinsed several times with ion-exchanged water until the solution reached a conductivity of <5 μS/cm^3^. The PEEK substrate with nanonetwork structures is denoted by PEEK-TNS. The processing procedure is illustrated schematically in Figure 7.

### 4.2. Sample Characterization

The surface morphologies of the PEEK, PEEK-Ti, and PEEK-TNS disks were observed using scanning electron microscopy (SEM) (S-4800; Hitachi, Tokyo, Japan). The three-dimensional surface topography and mean average surface roughness (Ra) were then obtained using a scanning probe microscope (SPM, Shimadzu, Tokyo, Japan). Cross-section processing of PEEK-Ti and PEEK-TNS was performed using a focused ion beam (FIB), and the cross-sectional morphology was observed at 50° using SEM. X-ray photoelectron spectrometry (XPS; PHI X-tool; ULVAC-PHI, Kanagawa, Japan) was used to evaluate the surface chemical states and elemental composition. The water contact angles were also measured using a contact angle measurement system (VS A2500 XE; AST Products, Billerica, MA, USA).

### 4.3. Cell Culture

The murine macrophage cell line RAW264.7 (EC91062702; KAC Co., Kyoto, Japan) was used to evaluate the inflammatory response to the samples in our research. RAW264.7 cells were maintained in high-glucose Dulbecco’s modified Eagle’s medium (DMEM; Nacalai Tesque, Inc., Kyoto, Japan) supplemented with 10% fetal bovine serum (FBS, Sigma-Aldrich, St. Louis, MO, USA) and 1% penicillin/streptomycin. Moreover, the cells were cultured at 37 °C with 5% CO_2_. Adherent cells were dislodged by gently passing a cell scraper upon reaching approximately 80% confluence. 

Rat bone marrow mesenchymal stem cells (rBMMSCs) were used to evaluate the osseointegration of the samples. Rat BMMSCs were isolated from the femurs of 8-week-old Sprague–Dawley rats (Shimizu Laboratory Supplies Co., Kyoto, Japan) and maintained in minimum essential medium (MEM, Nacalai Tesque, Inc., Kyoto, Japan) with 10% FBS and 1% penicillin/streptomycin [86]. The medium was replaced every third day. The confluent cells were subcultured by trypsinization, and cells at passages 3–5 were used for subsequent studies.

### 4.4. Inflammatory Response of Macrophages

#### 4.4.1. Cell Morphology and Viability of Macrophages

RAW 264.7 cells were seeded on the PEEK, PEEK-Ti, and PEEK-TNS surfaces in a 24-well plate at a density of 10^5^ cells/well. Prior to SEM (S-4800; Hitachi) observation, the cells on the samples were washed thrice with phosphate-buffered saline and fixed with 2% glutaraldehyde for 2 h. Furthermore, the cells were dehydrated through a series of ethanol concentrations (50%, 60%, 70%, 80%, 90%, and 99% anhydrous ethanol) using a critical point dryer (HCP-1; Hitachi, Tokyo, Japan). Thereafter, all samples were coated with platinum–palladium using an ion sputter machine (Ion sputter E-1030; Hitachi).

The viability of RAW264.7 on different samples after culturing for 3, 6, and 24 h was determined using the CellTiter Blue^®^ Cell Viability Assay (Promega, Madison, WI, USA). The cells were washed twice with PBS and stained with 300 μL of diluted CellTiter-Blue^®^ Reagent (50 μL CellTiter-Blue^®^ Reagent diluted in 250-μL PBS) at each time point. After 1 h of incubation, 100 μL reagent/well were transferred to a 96-well plate and examined using a spectrophotometer (SpectraMax M5; Molecular Devices, San Jose, CA, USA) at a wavelength of 560/590 nm.

#### 4.4.2. Polarization of Macrophages

The expression of M1 (TNF-α and IL-6)- and M2 (IL-10 and Arg-1)-related genes was analyzed using the real-time TaqMan reverse transcriptase-polymerase chain reaction (RT-qPCR) assay (Life Technologies, Carlsbad, CA, USA) after the RAW264.7 cells were seeded on different samples for 3 and 7 days. The relative gene expression levels of each sample were determined by the Ct method and normalized to the housekeeping gene glyceraldehyde 3-phosphate dehydrogenase (GAPDH).

#### 4.4.3. Osteogenic Gene Expression of Macrophages

The expression levels of VEGF, transforming growth factor beta 1 (TGFβ1), bone morphogenetic protein 6 (BMP-6), and oncostatin M (OSM) were analyzed by RT-qPCR to analyze the expression of osteogenic genes in macrophages of different groups. 

### 4.5. Cell Growth and Osteogenic Behavior of rBMMSCs Induced by Macrophages

#### 4.5.1. Preparation of Macrophage-Conditioned Medium (CM) for rBMMSCs

A RAW 264.7 cells suspension (10^5^ cells/well) was added to the PEEK, PEEK-Ti, and PEEK-TNS cells in a 24-well plate. The macrophage-conditioned medium was collected daily and centrifuged (2500 rpm at 4 °C) to obtain the supernatants. The culture medium (MEM containing 10% FBS and 1% penicillin/streptomycin with osteogenic supplements) was mixed with the obtained supernatant in a 2:1 ratio to obtain the conditioned medium (CM) and used for osteogenic activity experiments involving rBMMSCs.

#### 4.5.2. Osteogenesis and Osteoclastogenesis-Related Gene Expression of rBMMSCs

The rBMMSCs were seeded on a 24-well plate at a density of 4 × 10^4^ cells per well and incubated at 37 °C for 1 day. The culture medium was removed and replaced with CM. Osteogenesis-related genes, bone morphogenetic protein 2 (BMP-2), runt-related transcription factor 2 (Runx2), and bone carboxyglutamate (gla) protein (Bglap), as well as osteoclastogenesis-related genes such as osteoprotegerin (OPG), receptor activator of nuclear factor kappa-Β ligand (RANKL), and murine stem cell factor (MCSF) were analyzed using RT-qPCR after 3 and 6 days of incubation.

#### 4.5.3. Alkaline Phosphatase (ALP) Activity of rBMMSCs

The ALP activity of the rBMMSCs was measured using the ALP pNPP Liquid Substrate and enzyme-linked immunosorbent assay (ELISA) Kit (Sigma-Aldrich, St. Louis, MO, USA) after incubation for 7 and 14 days, and the DNA content of each sample was measured using the PicoGreen dsDNA Assay Kit (Thermo Fisher Scientific, Tokyo, Japan) based on the manufacturer’s protocol. The p-nitrophenol production was determined using a 96-well microplate reader (SpectraMax^®^ M5; Molecular Devices, Tokyo, Japan) at 405 nm, and the amount of ALP was normalized against the amount of DNA in the respective cell lysates.

#### 4.5.4. ECM Mineralization

The extracellular matrix (ECM) mineralization of the rBMMSCs was detected using a Calcium E-Test Kit (Wako Pure Chemical Industries, Tokyo, Japan). One milliliter of calcium E-test reagent and 2 mL of kit buffer were mixed and added to the cells, and the absorbance of the reaction was then measured at 612 nm using a 96-well microplate reader (SpectraMax^®^ M5; Molecular Devices) after culturing for 14 and 21 days.

### 4.6. Cell Growth and Osteogenic Behavior of rBMMSCs on Different Samples

#### 4.6.1. Cell Morphology and Viability of rBMMSCs on Different Samples

The RBMMSCs were seeded on the samples at a density of 4 × 10^4^ cells/well in a 24-well plate as the holder. The rBMMSCs on various samples were washed, fixed, dehydrated using the same treatments described in Section 4.4.1 after culturing for 24 h, and observed using the SEM thereafter. The cells on each disc were also counterstained with phalloidin (Alexa Fluor^®^ 488) and 4′, 6-diamidino-2-phenylindole (DAPI), as per the manufacturer’s instructions, and observed using a confocal laser scanning microscope (LSM700; Carl Zeiss, Japan, Tokyo).

The viability of the rBMMSCs cultured for 3 and 7 days on each sample was determined using the CellTiter Blue^®^ Cell Viability Assay.

#### 4.6.2. Osteogenic Behavior of rBMMSCs on Different Samples

The rBMMSCs were seeded on the PEEK, PEEK-Ti, and PEEK-TNS surfaces at a density of 4 × 10^4^ cells per well and incubated at 37 °C. The expression of the osteogenesis-related genes, including bone morphogenetic protein 2 (BMP-2), runt-related transcription factor 2 (Runx2), and bone carboxyglutamate (gla) protein (Bglap), were analyzed using RT-qPCR. The ALP activity was evaluated after culturing for 7 and 14 days. The extracellular matrix (ECM) mineralization of the rBMMSCs was determined using a Calcium E-Test Kit (Wako Pure Chemical Industries) after osteogenic induction for 21 and 28 days. 

### 4.7. Statistical Analysis

All quantitative results were expressed as the mean ± standard deviation. Statistical significance was analyzed using one-way analysis of variance (ANOVA) and Bonferroni’s post hoc test with SPSS software (version 20.0; IBM Corporation, Armonk, NY, USA). *p* < 0.05 was considered statistically significant. All experiments were conducted in triplicate.

## 5. Conclusions

In our research, the plasma sputtering deposition of the titanium and alkali treatment for PEEK was utilized to successfully produce a homogeneous porous titanate nanonetwork structure on its surface. The simplicity and cost-effectiveness of this technique may enable the mass production of biomaterials and implants. The cell experiments demonstrated that porous titanate nanostructures fabricated on PEEK significantly improved its biocompatibility, including the initial cell adhesion and proliferation. PEEK-TNS significantly modified the osteoimmune environment, including mediating of the M2 phenotype, suppressing the inflammatory response, and releasing osteoblast-promoting factors. The results of the macrophages and coculture with the osteoblasts experiments demonstrated that titanium nanostructures provided PEEK with beneficial immunomodulatory characteristics to inhibit the acute inflammatory response of macrophages and create a favorable osteoimmune microenvironment for enhancing osteogenesis effectively. Furthermore, the direct effects on osteoblasts cells on different samples were also detected, and the results showed that the nanostructure of PEEK-TNS greatly promoted osseointegration of the rBMMSCs. Therefore, the surface modification of PEEK with porous titanate nanostructures with enhanced osteoimmunomodulatory properties demonstrated in our research could be a highly suitable material for orthopedic applications.
